# Editorial: The cognitive neuroscience of empathy and its correlates

**DOI:** 10.3389/fnhum.2023.1321113

**Published:** 2023-10-31

**Authors:** Marina de Tommaso

**Affiliations:** Policlinico General Hospital, DiBrain Department, University of Bari Aldo Moro, Bari, Italy

**Keywords:** empathy, interoception, emotions, social relations, FMRI, EEG microstate, event-related potentials

Empathy is the ability to consciously share the affective state of another individual, who is recognized as the source of that state, and generates a similar perception in a “mirroring” condition (de Vignemont and Singer, 2006). In recent years, the biological basis of empathy has been emphasized, thanks to neuroimaging and electrophysiological methods in particular. This Research Topic contributes insights into the neuronal substrates of empathy, focusing on different cognitive, behavioral, and sensory aspects of shared experience.

The first article was published in May 2020 by Schaefer, Cherkasskiy, et al.. The study explored the involvement of the somatosensory system in the empathy network in healthy individuals, using FMRI. The experiment was based on the “hardness metaphor,” consisting of the cognitive and behavioral influence exerted by a rough tactile sensation in the social interaction. The 17 volunteers were requested to judge a crime and decide on a punishment, after a hard or soft tactile prime, exerting S1 activation. The more empathic subjects demonstrated weaker S1 activation after the hard tactile sensation, suggesting that empathy can reduce the tactile priming effects in the social domain. It seems an interesting and original contribution to the relationship between empathy, social interaction, and bodily sensations.

The study by Stoica and Depue, published in December 2020, explored the correspondence between interoception—i.e., the afferent processing of internal bodily signals that arise from visceral organs—and affective and cognitive empathy, using a paradigm of resting state functional connectivity (RSFC) and resting state—rsBOLD—variability related to brain networks underlying cognitive, affective empathy, and interoceptive awareness. They tested 26 young volunteers, using a non-social movie to activate the resting state network. The same subjects completed questionnaires for interoceptive awareness and empathy evaluation. The results showed a bidirectional behavioral relationship between empathy and interoceptive awareness, with different directions of correlation: negative for affective empathy and positive for cognitive empathy. Moreover, overall, empathy is related to our awareness of internal body changes, referring to specific brain area activation. The method the author used was also innovative in terms of its translational application in patients with severe empathy deficits and a low level of cooperation, e.g., autism disorders.

The study by Schaefer, Joch, et al., published in February 2021, returned to the relationships between the somatosensory system and empathy, exploring in particular the correlation between the capacity to share the other's experience and tactile accuracy. Their experiment was performed with a large group of healthy individuals (95 participants). It was based on behavioral and psychophysic responses, obtained with standardized tests for sensitive accuracy and empathy. The authors found that tactile accuracy was positively correlated with an empathetic attitude. The study seems relevant in terms of case numbers and the replicability of the methods and finally confirms that the capacity to understand another person's feelings depends on the ability to know their own bodily sensations.

Zhang et al., in a following study published in June 2021, explored the relationship between individual empathy and the expression of disgust, using behavioral tests and EEG parameters—the so called microstates. The EEG functional microstate is a method that can display brief sequences of approximately 100 msec, which proceed in rapid sequence, designing a no-task resting condition into cortical source activation. Using this strongly time-related functional method and behavioral tests, the authors found that disgust and empathy scores were positively correlated and that the modality of transition between microstates had a correspondence with empathy. Despite the limitation of the EEG method, which is not spatially accurate, the large case numbers and the correct use of behavioral scores, as well as the contribution of a reliable method to detect time-related cognitive brain correlation, render the results interesting and of potential interest in the area of a relevant contribution in the psychophysiological context.

The most recently published article by Fan et al. used an event-related potentials paradigm focusing on P3 and late positive responses to detect emotional and arousal reactions under the vision of different situations of another person's pain. The task was preceded by a standardized game that reproduced situations of social exclusion or inclusion. The 45 college students demonstrated that the condition of social exclusion simulated in the game reduced in some way the late arousal involvement in another person's pain, expressed by the late positive potential. These results add new contributions in terms of complex relationships among different interpersonal interactions in a social context.

In summary, this Research Topic provided relevant contributions about the cognitive compounds of empathy in healthy subjects, exploring the relationships with bodily self-interoceptive sensations, emotions, and social interactions. Studies examined rigorous methods of behavioral scores and brain functional analysis, further underlining the importance of spatial and time-related techniques in the psychophysiology domain, with potential and the required application in clinical neurosciences.

## Author contributions

MT: Conceptualization, Writing – original draft.
